# The relationship between acoustic habitat, hearing and tonal vocalizations in the Antillean manatee (*Trichechus manatus manatus*, Linnaeus, 1758)

**DOI:** 10.1242/bio.013631

**Published:** 2015-09-04

**Authors:** Mario Rivera Chavarría, Jorge Castro, Arturo Camacho

**Affiliations:** 1 Centro de Investigaciones en Tecnologías de la Información y Comunicación, Universidad de Costa Rica Sede “Rodrigo Facio Brenes” Montes de Oca, San José 2060, Costa Rica; 2 Escuela de Ciencias de la Computación e Informática, Universidad de Costa Rica, Sede “Rodrigo Facio Brenes” Montes de Oca, San José 2060, Costa Rica

**Keywords:** Acoustics, Hearing, Manatee, Vocalization

## Abstract

The Antillean manatee (*Trichechus manatus manatus*) is an endangered marine mammal that inhabits the Caribbean Sea and riverine systems in Central America. Their acoustic behavior is relevant for individual identification, mating and parental care. Manatees produce tonal sounds with highest energy in the second harmonic (usually 5 kHz), and their audiogram indicates sensitivity from 0.3 kHz to 90 kHz with lowest thresholds in the 16 to 18 kHz range. We recorded manatees in the San San River, a highly polluted riverine system in Panama, using a stereo array. Frequency transmission experiments were conducted in four subhabitats, categorized using riverine vegetation. Incidental interactions of manatees and small motorboats were examined. Acoustic transmission was linearly related to tonal vocalization characters: correlations were stronger in freshwater than in transition and marine environments. Two bands, 0.6 to 2 kHz and 3 to 8 kHz, attenuate similarly in all subhabitats, and these bands encompass *F*_0_ (tone) and peak frequency respectively of manatee tonal calls. Based on our data we conclude that frequency transmission depends mainly on river depth and bottom characteristics, also motorboat sounds mask signals from 3.5 kHz to 8 kHz, which overlaps the peak frequency of tonal calls. In spite of differences between acoustic transmission in subhabitats of the San San River, manatees utilize bands that transmit efficiently in all subhabitats.

## INTRODUCTION

Manatees produce sounds in many behavioral contexts including social cohesion, reproduction, aggression, danger, and parental care (Sousa-Lima et al., 2002, 2008; Nowacek et al., 2003). Their productions can be tonal or atonal (Mann et al., 2006) and have sound pressure levels around 100 dB re: 1 μPa at 1 m (Phillips et al., 2003). For tonal emissions, fundamental frequency is around 2 kHz, and the second harmonic usually contains the highest energy (Gerstein et al., 1999; Colbert et al., 2009). Calls with frequencies above 20 kHz are produced during parent-offspring interactions (Gerstein et al., 1999) mother-calf pairs are known to have a high vocalization rate, and stereotypical differences in fundamental frequency (Sousa-Lima et al., 2002). These tonal characteristics are similar in manatee (*Trichechus manatus spp.*) populations from Belize and Florida. Anatomical, or physiological divergences are unlikely be present between these populations (Nowacek et al., 2003; Ketten et al., 1992).

Manatees are most sensitive to the range from 16 kHz to 18 kHz (Gerstein et al., 1999; Gaspard et al., 2012) but are capable of hearing frequencies up to 90.5 kHz (Gaspard et al., 2012). Manatees have one of the lowest critical ratios reported in mammals, likely an adaptation to noisy environments (Gaspard et al., 2012).

Auditory capabilities of manatees are closely related to their abilities for sound source localization. In general, mammals use two mechanisms to detect sound sources: inter aural timing difference (ITD) and inter aural level differences (ILD) (Colbert et al., 2009). For manatees there is strong evidence to suggest the use of both: ILDs are likely to be used with short wavelength signals (high frequencies), whereas ITD can be better for sounds with larger wavelengths. Recent research has shown that ILDs show an anomalous response in the 0.2 kHz to 1.5 kHz band (Colbert et al., 2014). There is also a strong directional response around 5 kHz (Colbert et al., 2009).

In turbid environments, with limited visual cues, sound is the most efficient communicational channel (Bauer et al., 2003). Bottom type, temperature, moon phase, tides, salinity, pollution, environmental flow and physical barriers can all influence acoustic propagation (Rogers and Cox, 1988). In an ecologically altered estuarine system, environmental flows can change water properties suddenly, and it is difficult to generate accurate mathematical models of physical-chemical factors (Allan and Castillo, 2007; Tharme, 2003) and acoustic propagation (Miksis-Olds and Miller, 2006). Miksis-Olds and Miller (2006) have shown that habitats where manatees are frequently associated as marine grasses have high transmission loss above 2 kHz which may affect hearing and call spectra (Miksis-Olds and Miller, 2006; Miksis-Olds et al., 2007; Gerstein, 2002; Ey and Fischer, 2009).

Environmental transmission characters have been shown to shape hearing physiology and vocal behaviour in other mammals, birds, anurans and fishes (Ey and Fischer, 2009; Brown and Waser, 1988; Lugli et al., 2003). In general, appears that aquatic animals use frequencies from quieter bands to optimize communication (Lugli et al., 2003).

We asked ourselves how does the environment shape manatees hearing physiology and vocal characteristics. To answer this question, we determined the habitats acoustic characteristics and interpolated it with vocalization data and hearing physiology. This was carried out in a manatee habitat at the San San River wetland in Panama (Sue et al., 1990).

**List of symbols and abbreviations**

AR: Autonomous recorder
dB: Decibels
*F*_0_: Fundamental frequency
*F_s_*: Frequency sampling
FT: Frequency transmission
ILD: Inter aural level difference
ITD: Inter aural timing difference
SC: Sensitivity curve

## RESULTS

**Fig. 1.**
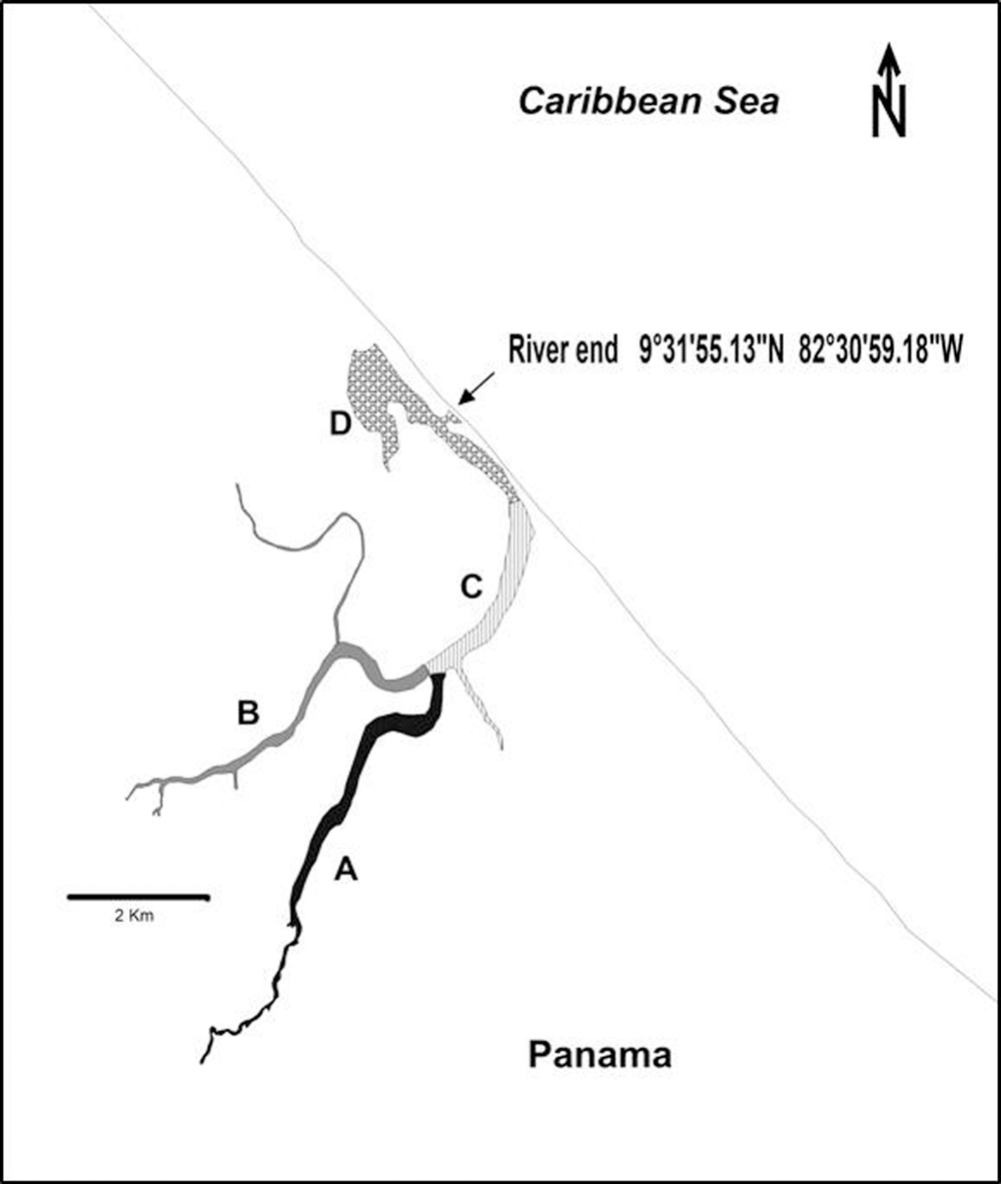
**Schematic map of the study area with the subhabitats marked with letters.**

**Fig. 2.**
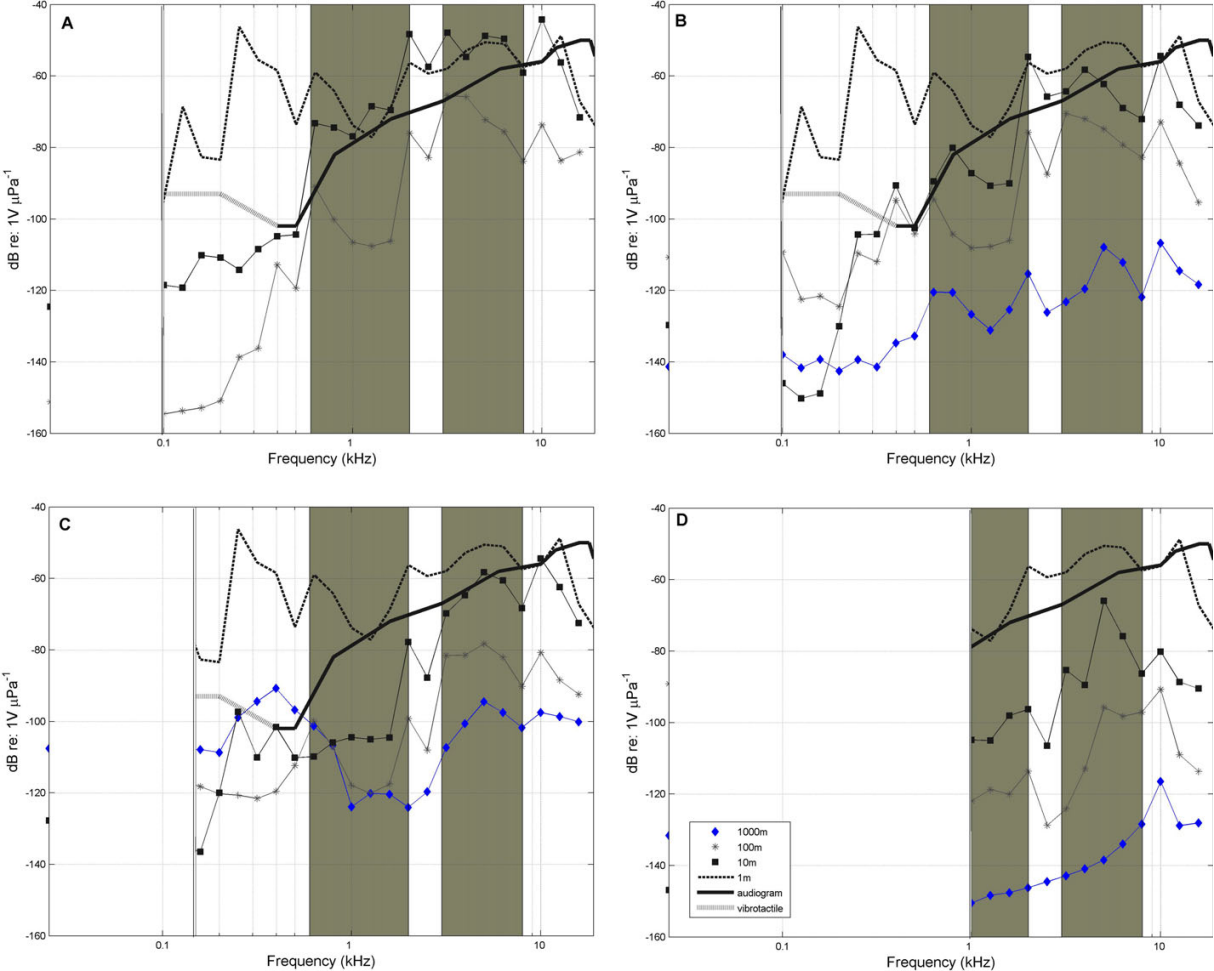
**Plots showing the averages of frequency transmission experiments.** Results of frequency transmission experiments in different subhabitats A–D. Location A is a freshwater site which riverine vegetation is dominated by grasses for agricultural use. It does not show the 1000 m curve due to wave physical limitation caused by sinuosity. Location B is fresh water environment with different riverine vegetation (mangrove and ferns). Location C is a transition zone populated mainly by mangroves. Location D is a marine coastal lagoon with sedimentary acoustic barriers with very limited transmission. All the sites share frequency propagation characteristics highlighted by shadowed areas. Frequency cutoffs were estimated using Eqn 1. Vibrosense related curve was plotted with a discontinuous line in order to preserve Gerstein et al's. manatee audiogram (Gerstein et al., 1999), but it is irrelevant for analysis purposes. Vertical double line shows the frequency cutoff.

Comparison between the sweep signal at 10 m and greater distances indicated that transmission was greater in freshwater and transition environments (with depths ranging between 9 m to 15 m and 15 m to 20 m) than in the coastal lagoon (with depths ranging from 1 m to 1.5 m) (Figs 1 and 2). Similar attenuation occurred in two bands in all subhabitats: 0.6–2 kHz and 3–8 kHz. From 0.6 kHz to 2 kHz signal loss is 0.4 dB/m (±0.1 dB/m) in the first 10 m, 0.04 dB/m (±0.2 dB/m ) in the following 100 m and 0.004 dB/m (±0.002 dB/m) in the last 1000 m. In the band from 3 kHz to 8 kHz power loss is significantly lower than in the first mentioned band averaging 0.2 dB/m (±0.01 dB/m). These similarities are constant across all sites except for location A (Fig. 2A) and location C (Fig. 2C). In location A signal was not received farther than 100 m away at all. In location C, at 1000 m signal was only received in frequencies around 10 kHz. Manatees show increased sensitivity from 0.5 kHz to 18 kHz (Gerstein et al., 1999), coinciding with the bands that propagate maximally (for the freshwater subhabitat A: *r*^2^=0.65, *P*=0.05, Fig. 2A; for the freshwater subhabitat B: *r*^2^=0.67, *P*=0.05, Fig. 2B; for the transition subhabitat C: *r*^2^=0.54 *P*=0.05, Fig. 2C; and for the marine subhabitat: *r*^2^=0.54 *P*=0.05, Fig. 2D). Subhabitats do have different transmission characteristics, according to Eqn 1. Subhabitats A and B (2A,B) show a frequency cutoff of 0.1 kHz, while subhabitat C (Fig. 2C) shows a frequency cutoff of 0.15 kHz and subhabitat D (Fig. 2D) shows not to transmit frequencies below 1 kHz.

**Fig. 3.**
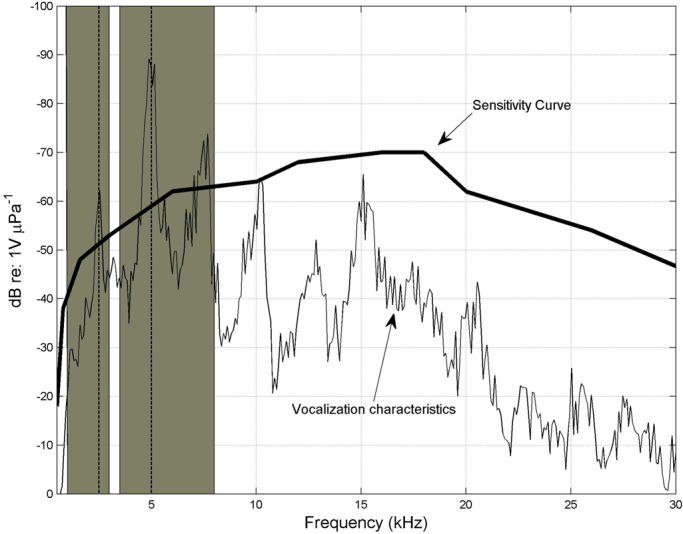
**Vocalization average characteristics weighted with sensitivity curve.** Highlighted areas correspond to the bands that show similar power loss in the frequency between all transmission experiments. Average *F*_0_ (2.9 kHz) and peak frequency mode (5 kHz) are marked with discontinuous vertical lines.

For manatee tonal productions, average tone is around 3 kHz with a range between 0.7 to 8.1 kHz. Peak frequency average is around 6 kHz, with a mode in 5 kHz and a range between 2 kHz and 15 kHz. The average duration is 362 ms and does have high variability (±114 ms) (Table 1). The average spectra was plotted (Fig. 3), and the resultant curve was compared with the SC (sensitivity curve) showing a strong Pearson correlation (*r*^2^=0.67, *P*=0.05).

**Fig. 4.**
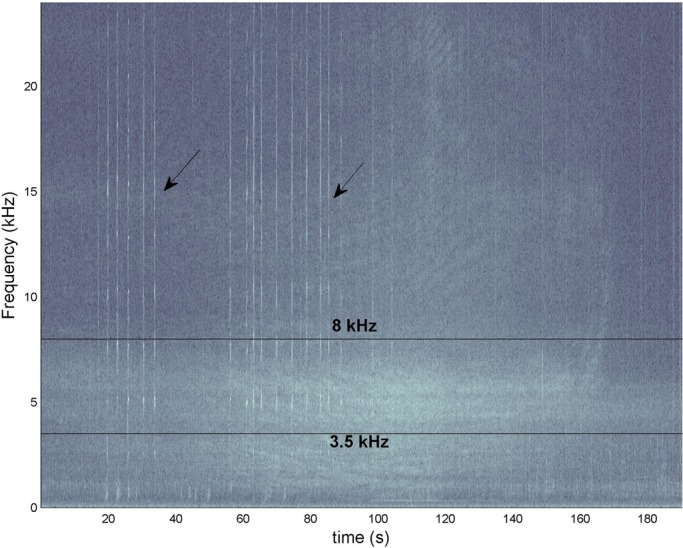
**Representative spectrogram showing tonal vocalizations and masking noise from distant motorboats (more than 25 m from manatees).** The band from 3.5 kHz to 8 kHz is delimited by two lines. Manatee tonal vocalizations are marked with arrows.

Call rate disruption in boat-manatee distances farther than 25 m was not evident. Manatees maintain an average call rate of 20 tonal calls per minute (*n*=12). When the motor sound overwhelms the spectra, calls cease and any other sign such as bubbles and movement is not evident. It is notable that boats from a distance higher than 50 m mask the band from 3.5 kHz to 8 kHz. Fig. 4 shows a representative scenario of an encounter of a vocalizing manatee and a motor boat. Tonal vocalizations are marked with arrows.

## DISCUSSION

The San San river habitat holds four acoustic subhabitats with different frequency transmission characteristics. The upper river system, consisting of fresh water, exhibits less signal attenuation especially in low frequency sounds. Then, a transition zone exhibits a higher level of marine noise compared to freshwater subhabitats. Finally, a shallow marine zone ending in a coastal lagoon has very low range frequency transmission characteristics. Spectra from the four zones are different and can be easily differentiated. Sound transmission in the habitat is influenced by estuarine processes and may be masked by biological activity caused by snapping shrimps, turtles, otters, and soniferous fish. Estuarine sedimentary cumulative effects are significant environmental acoustic modelers. For example, the river end is before a coastal lagoon, in consequence the sediments are accumulated. This is the limit between two acoustic subhabitats: transition and marine. In all subhabitats biological sound daily dynamics are a major agent of change, especially in the evening, when fish sounds overwhelm spectra in marine and transition zones. Riverine vegetation does have a direct influence on the underwater river soundscape (Fig. 1). In spite all of these factors, spectra in the subhabitats do show stereotypical frequency attenuation characteristics in the 0.6 kHz to 2 kHz and 3 kHz to 8 kHz range.

The FT data indicate distances that a broadband sound with similar characteristics with manatee vocalizations can be emitted and received based on attenuation. There is a significant correlation between tonal vocal behaviour, hearing physiology of manatees and typical frequency transmission characteristics of the environments.

*F*_0_ is situated in a band where frequency power attenuation is relatively high. Peak frequency however, is located in a band that is efficiently transmitted. Although our results are difficult to compare, because of our main point is the SC weighting, our findings are partially supported by a previous study (Miksis-Olds and Miller, 2006) in a similar environment. In other species where the auditory system evolved completely underwater such as toadfishes, their characteristic whistles do not propagate for long distances. Toadfishes vocalize in certain frequency bands for long range detection and localization, these bands are used with chorusing, individual communication in contrary appears to be effective at ranges of few meters (Fine and Lenhardt, 1983). In the case of manatees they do show increasing sensitivity in bands with optimal propagation for general feature recognition (gender, size, age) (Sousa-Lima et al., 2002) and positional information (Colbert et al., 2009). This does not mean that information encoded in other bands (such as frequencies with manatees’ highest sensitivity, from 16 kHz to 18 kHz) is irrelevant. It means that individual recognition occurs at a short range, since high frequency intensity decrease more rapidly close to the source (Mohajer et al., 2014). This characteristic seems to be recurrent in other shallow water species of other animal groups (Ghahramani et al., 2014).

Peak frequency for the Belize population was from 3 kHz to 5 kHz and 5 kHz in Crystal River (Florida, USA) (Nowacek et al., 2003). These results are similar to the ones found in this study, confirming their relevance. Regarding other relevant bands, tone (*F*_0_) values (Table 1.) seem to be more similar to the Florida manatee population (2.8 kHz) than to the one in Belize (3.7 kHz). This difference is due to environmental conditions rather than phylogeny (Ey and Fischer, 2009; Ketten et al., 1992), although the second possibility and the effects of disruptive effects of river environmental flows should be addressed.

**Table 1.**
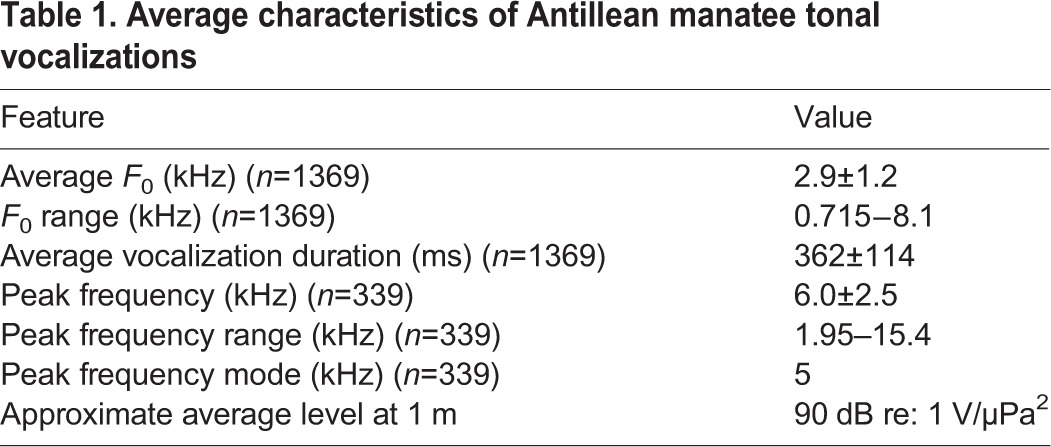
**Average characteristics of Antillean manatee tonal vocalizations**

Distant boat noise (more than 25 m away from the receiver) masks a band from 3.5 kHz to 8 kHz necessary for directional responses (Fig. 4) (Colbert et al., 2009). ITD's are most likely used for sound source localization (Colbert et al., 2009). Therefore manatees are particularly vulnerable in reverberant environments such as channels where inter-aural envelope coherence is degraded (Monaghan et al., 2013). Recently Colbert et al. (2014) determined that manatees are capable of detecting sound sources with longer wavelengths than their intermeatal or intercochlear distance; they show responses to frequencies from 0.2 kHz to 1.5 kHz. This band overlaps with strong river-flow sounds (Lugli et al., 2003). Therefore, in the current study scenario, ITDs will be important for localization. Also, low-frequency cuttoffs, a function of the shallow depths, can modify signals and cut frequencies that in this study are from 0.1 kHz to 1 kHz. Motorboats overlaps a band specifically important for determining directionally (Gerstein et al., 1999), and this can affect manatees’ avoidance behaviour in small boat routes. Vulnerability increases at night when natural sounds overwhelm spectra in marine and transition areas.

Water level loss in estuaries such as the San San River can increase shallow areas where communication is limited. Therefore climate change could mimic the effects of environmental flows caused by hydroelectric and agricultural projects, limiting animal communication and consequently affecting the population dynamics. Acoustic communication transmission depends on the propagation characteristics. These characteristics are originally defined by the river natural environmental flow and later affected by pollution. The effect of pollution should be addressed.

## MATERIALS AND METHODS

The San San River is a protected wetland by the Secretariat for the Convention on Wetlands (RAMSAR). It is an estuarine system located in Panama, Central America (Fig. 1). Despite of its conservation status, fecal and agrochemical pollution are in levels considered dangerously high for humans. This was reported in a technical document by the University of Panama Chemical Science Department (Caballero et al., unpublished). Swimming- related activities were avoided for health security reasons.

Initially, habitat was classified in categories according to the vegetation characteristics: freshwater, transition, and a coastal lagoon with marine influence. The vegetation does have a direct influence on river morphology and riverside slope characteristics (Allan and Castillo, 2007).

The fieldwork detailed in this work was carried out from January to November 2013, 15 days per month. It consisted of characterizing manatees’ habitat acoustics and vocalizations as detailed in the following sections.

### Habitat characterization

To characterize the habitat acoustics recordings were made in the three subhabitats with six autonomous recorders deployed along the river. This was carried out to confirm that those present different underwater acoustic characteristics. To determine this, six autonomous recorders were deployed simultaneously along the river. The deployment was done once a month for 4 months, from March to June, 2013. Autonomous recorders (ARs) were modified mini RUDARS (Cetacean Research^®^, Seattle, Washington, USA). Duty cycle ARs were adapted by attaching microprocessors (ARDUINO^®^ mini, Ivrea, Italy) to the H1 recorders (Zoom^®^, Chiyoda-ku, Tokyo, Japan). ARs were set to record for one minute every ten minutes, for 21 days. Synchronization was made using the device clock and an initial impulsive event was made with a metallic sound.

Based on early results from autonomous recorders, it was determined that fish chorusing (mostly *Cynoscion jamaiscensis*), snapping shrimp, and unidentified species of toadfishes, overwhelm spectra of marine influenced areas during the evening (16:00 to 06:00 h). This completely masks manatees’ characteristic sound. Therefore, fieldwork was usually carried out only during times of non targeted biological low activity, from 06:00 to 16:00 h.

### Frequency transmission experiments

It is worth to note that, within freshwater subhabitats, there were differences in riverine vegetation composition. Hence, we carried out the procedure on each of those two separately. FTs were done instead of applying models since those appear to be inaccurate for shallow environments (Miksis-Olds and Miller, 2006).

The sweeps were recorded using a CR1 hydrophone calibrated at −198.51 dB 1 V re:1 V 1 μPa^−1^ (Cetacean Research^®^), a dual pre-amplifier (ART^®^, Niagara Falls, New York, USA), and a H4n^®^ recorder (Zoom^®^). A reference sweep (9 s at 1 m, from 0.02 kHz to 19 kHz) with an intensity of 110 dB at 5 kHz (salt water, 34 mg/l, at 22°C) was made in the Naos Marine Laboratory at the Smithsonian Tropical Research Institute in Panama. Since the speaker (Clark Synthesis^®^, Littleton, Colorado, USA) cannot maintain uniform power across the sweep, the 110 dB intensity was only obtained at 5 kHz, which is actually the reported manatees’ peak frequency. The sweep, with identical equipment and settings, was applied at each of the four localities specified earlier. In the field, we recorded the played back sound at 10 m, 100 m and 1000 m from the stationary source. Sweep was presented three times per location. An artificial sweep was used for resolution purposes and mainly because of the difficulty to find a broadly representative tonal call. Variance among call characteristics is very high as showed in the summary of the statistics of the samples. The data obtained from each frequency transmission experiment was filtered by a 1/3 octave filter bank. Then, the power spectral density was determined using the Welch method (Welch, 1967), using a 128 samples window with no overlap. The intention of this procedure was to obtain general and stereotypical characteristics, by obtaining bands with uniform power attenuation. We did not measure the physical-chemical characteristics of the water.

Sweep playback was applied only when needed, to avoid possible disturbance or damage to local aquatic life. This procedure was not possible to carry out in the field for distances shorter than 10 m due to river current, swimming avoidance and low maneuverability of the stationary source.

### Manatee localization and recording

Vocalizing manatees were located using a two hydrophone array, kayaking a 24 km transect daily, completing 369 field hours. The methodology consisted on using a mobile stereo array to locate them using human interaural level difference capabilities. The array was mounted in the kayak and and its design considered the minimum distance between hydrophones for accurate detection of average manatees peak frequency (5 kHz) (Colbert et al., 2009; Gaspard et al., 2012; Gerstein et al., 1999; Nowacek et al., 2003), assuming an underwater sound speed of 1500 m/s (Carlton, 2012). Distance between hydrophones was 86 cm and minimum effective distance for interaural detection is 30 cm. Once the animal (animals) was (were) located, the distance source-hydrophone was estimated using surface indications of manatee presence, such as movement sounds, vocalizations and bubbles. This allowed calculation of the approximated source level. Manatee productions were recorded using the array for location purposes but their level was estimated using the channel that was nearer to the source. At least 9 individuals were recorded.

During manatee-motor boat interactions, recordings were longer (usually more than 5 min, but not more than 8 min). After that, transects were continued. Data from these recordings was processed using MatLab^®^ spectrogram function with a 1024 samples window and no overlap.

Data from the West Indian manatee audiogram was used to transpose with the frequency transmission data and tonal characteristics. In order to know what frequencies are efficiently transmitted in the subhabitats, depths were measured for all of the river. Frequency cutoff (*F_c_*) of the sites was determined using the following equation (Rogers and Cox, 1988):
(1)
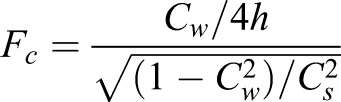

where *c_w_* is the speed of sound (m/s), *h* is average depth (m), and *c_s_* is the speed of sound in the sediment (m/s). Sound speed in the sediment was estimated by sampling from the bottom, and comparing its characteristics with predefined values (Rogers and Cox, 1988).

For analysis purposes, Antillean manatee tonal sounds were isolated from recordings. Only tonal sounds were used since fundamental frequency and duration are the most important features for communication (Sousa-Lima et al., 2002, 2008). Also, nonlinear sounds were excluded since big fishes such as tarpoons and catfishes, river turtles and mammals such as otters produce atonal sounds which are very similar to the ones presented by the manatees.

A total of 1369 isolated sounds were obtained. Each sound was initially analyzed using the pitch estimator SWIPE (Camacho and Harris, 2008). If the sample was noisy, *F*_0_ was estimated manually using its spectrogram. Peak frequency was estimated using Matlab^®^ peak detection algorithm (*findpeak*). If the sample was noisy, peak frequency was also estimated manually using its spectrogram. Other features such as duration, frequency range and relative intensity were registered manually from wave and spectral data. Since there is not much literature detailing the occurrence of frequencies above 22 kHz, recordings were made at a sampling frequency of 44.1 kHz using 32 bits per sample. During field work, the recurrent presence of ultrasonic components was evident. Hence, *F_s_* was incremented to 96 Hz. Therefore, peak frequency statistics as well as the average approximate level were estimated using 339 samples, which were taken at 96 kHz 32 bits.

### Data correlation

Comparisons of FT experiments and audiological information obtained from a sensitivity curve, which is defined as the inverted sign magnitude of the manatees audiogram (Gerstein et al., 1999) were carried out. Gerstein et al. (1999) audiogram is more appropriate for this work since we do not use equipment able to measure high frequencies as was done in a later study (Gaspard et al., 2012). In any case, both audiograms are very similar. Inverted audiogram (sensitivity curve) is a measure of how sensitive the animal is to certain frequency bands, so can be considered as a weighting curve. FTs at 10 m were averaged and signal spectra were calculated for each locality. Then, the lower limit for correlation using frequency cut offs was calculated and interpolated with the hearing sensibility curve points corresponding to 1/3 octave filter of the FT at 10 m. Finally, we calculated Pearson correlation coefficient between sensitivity curve and the mentioned FT for each location.

To determine the correlation between the hearing sensitivity and average vocalization a 30th order moving average (approximately 2.8 kHz of neighborhood) was firstly applied to the average vocalization spectrum to obtain a smoother signal to compare with.
